# A Role of the *FUZZY ONIONS LIKE* Gene in Regulating Cell Death and Defense in Arabidopsis

**DOI:** 10.1038/srep37797

**Published:** 2016-11-29

**Authors:** Arianne Tremblay, Savanna Seabolt, Hongyun Zeng, Chong Zhang, Stefan Böckler, Dominique N. Tate, Vy Thuy Duong, Nan Yao, Hua Lu

**Affiliations:** 1 Department of Biological Sciences, University of Maryland Baltimore County, 1000 Hilltop Circle, Baltimore, MD 21250, USA; 2 State Key Laboratory of Biocontrol, Guangdong Provincial Key Laboratory of Plant Resources, School of Life Sciences, Sun Yat-sen University, Guangzhou 510275, P.R. China; 3 Institut für Zellbiologie, Universität Bayreuth, Bayreuth 95440, Germany

## Abstract

Programmed cell death (PCD) is critical for development and responses to environmental stimuli in many organisms. FUZZY ONIONS (FZO) proteins in yeast, flies, and mammals are known to affect mitochondrial fusion and function. Arabidopsis FZO-LIKE (FZL) was shown as a chloroplast protein that regulates chloroplast morphology and cell death. We cloned the *FZL* gene based on the lesion mimic phenotype conferred by an *fzl* mutation. Here we provide evidence to support that *FZL* has evolved new function different from its homologs from other organisms. We found that *fzl* mutants showed enhanced disease resistance to the bacterial pathogen *Pseudomonas syringae* and the oomycete pathogen *Hyaloperonospora arabidopsidis*. Besides altered chloroplast morphology and cell death, *fzl* showed the activation of reactive oxygen species (ROS) and autophagy pathways. *FZL* and the defense signaling molecule salicylic acid form a negative feedback loop in defense and cell death control. *FZL* did not complement the yeast strain lacking the *FZO1* gene. Together these data suggest that the Arabidopsis *FZL* gene is a negative regulator of cell death and disease resistance, possibly through regulating ROS and autophagy pathways in the chloroplast.

Programmed cell death (PCD) is an integral part of proper development and responses to environmental stimuli of many organisms^1^,^2^. Apoptosis and autophagy are the two main forms of PCD in animal cells. Although not exhibiting the classic apoptosis, plant cells can undergo autophagy, involving the formation of membrane-bound vesicles to sequestrate cellular content for degradation and recycling and to determine the survival of cells^3^,^4^,^5^. Many core autophagy components, the autophagy-related (*ATG*) genes, are identified in plants and they share similar sequence and function with their homologs in other organisms^6^. Autophagy plays a critical role in plant development and nutrient recycling. Recent studies have also implicated a role of autophagy in plant defense against pathogens, in particular determining PCD in the infected plants^7^,^8^. However, many questions still remain regarding how autophagy affects PCD under defense conditions.

Besides pathogen-induced cell death, some mutant plants exhibit constitutive cell death in the absence of pathogen infection. Such mutants are collectively called lesion mimic mutants. These plants often have enhanced disease resistance, strengthened cell walls, express more defense-related genes, and accumulate faster and/or more defense-related molecules, such as salicylic acid (SA) and reactive oxygen species (ROS)^9^,^10^. Functional analyses of some corresponding genes of the lesion mimic mutants have revealed multiple pathways leading to PCD, among which the chloroplast appears to be an important source for pro-death signaling^10^. Although important for pathogen induced PCD in plants, the role of autophagy has not been well understood in plants with autoimmune defects. Mutations in some *ATG* genes that were associated with early senescence and cell death phenotypes indeed suggest a critical role of autophagy in cell death control observed in some lesion mimic mutants^11^.

The *FUZZY ONIONS (FZO*)*-LIKE (FZL*) gene of Arabidopsis is a single copy gene encoding a GTPase-domain containing protein in the dynamin superfamily. *FZO* genes in yeast, flies, and mammals were shown to affect mitochondrial fusion. Disruption of these *FZO* genes could lead to mitochondrial fragmentation and dysfunction^12^,^13^,^14^,^15^,^16^,^17^,^18^. An Arabidopsis *fzl* mutation was previously shown to confer cell death and altered chloroplast morphology, associated with higher accumulation of some defense related molecules^19^. However, it is unknown how the Arabidopsis *FZL* gene affects cell death and whether it regulates mitochondrial function and plant resistance to pathogens.

We identified a lesion mimic mutant in the background of a transposon insertional mutant for the phosphate transporter gene *PHT4;1*^20^. Positional cloning revealed that the lesion mimic phenotype was not associated with the *pht4;1* mutation, but was due to a mutation in the *FZL* gene. Besides its known role in affecting cell death and chloroplast morphology, we report here new function of the *FZL* gene in regulating plant defense and autophagy. We found that *fzl* mutations conferred enhanced disease resistance to the bacterial pathogen *Pseudomonas syringae* and the oomycete pathogen *Hyaloperonospora arabidopsidis (Hpa*). *fzl-*conferred cell death was associated with the activation of the autophagy and ROS pathways and was dependent on the key defense signaling mediated by SA. Our data also support a reciprocal negative regulation between FZL and SA signaling. Although being the only close homolog of Arabidopsis to the mitochondria-localized FZO proteins from yeast, flies, and mammals, a functional FZL-YFP chimeric protein was localized the chloroplast outer membrane but not the mitochondria of Arabidopsis cells. Thus, Arabidopsis *FZL* gene may have evolved new function different from its other homologs. Consistent with this idea, we found that neither *FZL* nor an *FZL* variant rescued the yeast strain lacking the *FZO1* gene. Together, our study suggests that the *FZL* gene is a negative regulator of PCD and disease resistance to biotrophic pathogens in Arabidopsis, possibly acting through the autophagy pathway.

## Results

### FZL is a negative regulator of disease resistance to biotrophic pathogens in Arabidopsis

**Figure 1:**
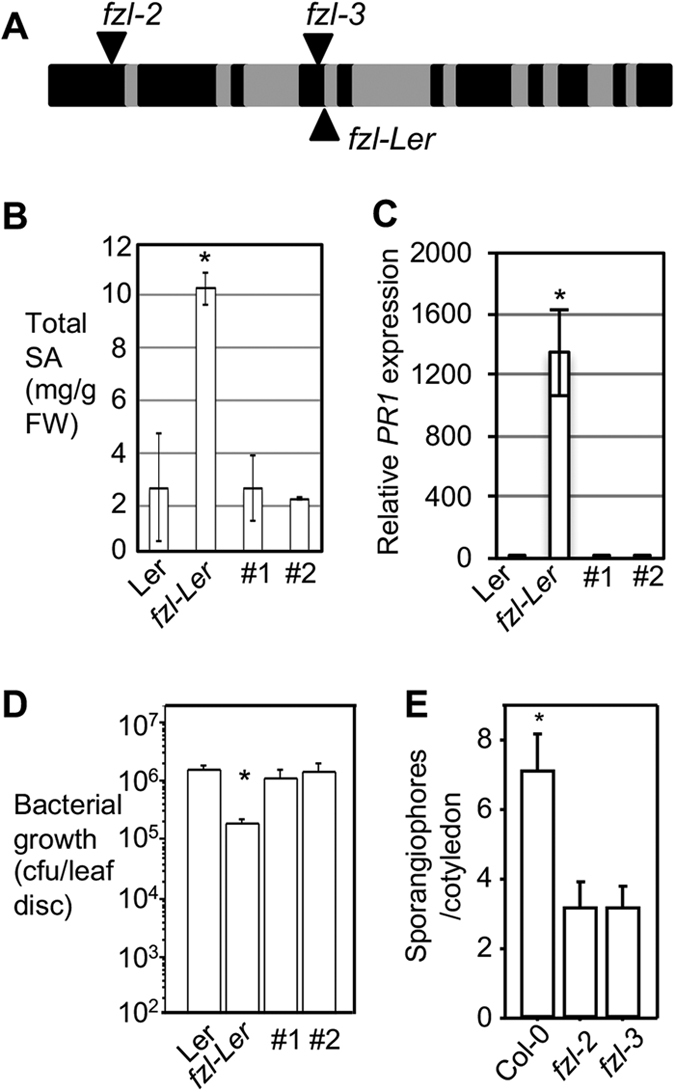
*fzl* mutations confer enhanced disease resistance to bacterial and oomycete pathogens. (**A**) The *FZL* gene structure and *fzl* mutant alleles. Exons are indicated in black and introns in grey. (**B**) SA quantification. Total SA (glucosylated form) was extracted from the indicated plants and quantified with an HPLC instrument. (**C**) Expression of *PR1* analyzed by qRT-PCR. (**D**) Bacterial growth assay. Plant leaves were infiltrated with virulent *P. syringae* pv. tomato strain DC3000 (OD_600_ = 0.0004). Leaf discs were taken 3-day post infection for bacterial growth measurement. (**E**) *Hpa* Noco2 infection assay. Seven-day-old seedlings were spray-infected with *Hpa* Noco 2. Sporangiophore production in cotyledons of each genotype was counted at 7 dpi. Data represent the average number of sporangiophores from 50 seedlings with SEM. Statistical analysis was performed with Student’s t-test (StatView 5.0.1). The asterisk indicates significant difference between the labeled sample and other samples (P < 0.01). These experiments were repeated at least two times and similar results were obtained.

We isolated a lesion mimic mutant in the background of the phosphate transporter mutant called *pht4;1-2* (GT_5_110509; a *Landsberg erecta* allele)^20^. The lesion mimic phenotype progressed more severely as leaves became more senescent (Fig. S1A). This phenotype was not co-segregated with the transposon insertion in *PHT4;1* and kanamycin resistance. To identify the responsible gene, we crossed this mutant to Columbia-0 (Col-0) and conducted map-based cloning with 1108 recombinant F_2_ lines to narrow down the mutation to a 50 kb region on chromosome 1. Further sequencing of the coding fragments in the region identified a one base pair substitution (G > A) in the *FZL* gene (At1g03160), which encodes a protein in the dynamin superfamily with demonstrated roles in regulating chloroplast morphology and cell death^19^,^21^. The mutation, previously called *fzl-Ler*, disrupts the 5′ end exon-intron junction of intron IV (Fig. 1A and ref. 19).

Using a chimeric construct *FZL-YFP*, created by fusing the *FZL* cDNA translationally at the 3′ end with the *YFP* reporter under the control of the *CAMV 35S* promoter, we were able to complement phenotypes conferred by *fzl-Ler*, including cell death, high SA accumulation, and high expression of SA marker gene *PR1* (Fig. 1B,C and S1B-S1D). To test if *FZL* regulates disease resistance, we infected *fzl-Ler* with *Pseudomonas syringae* pv. *tomato* strain DC3000 (DC3000). The mutant showed more resistance than Ler, which was rescued by the *FZL-YFP* transgene (Fig. 1D).

**Figure 2:**
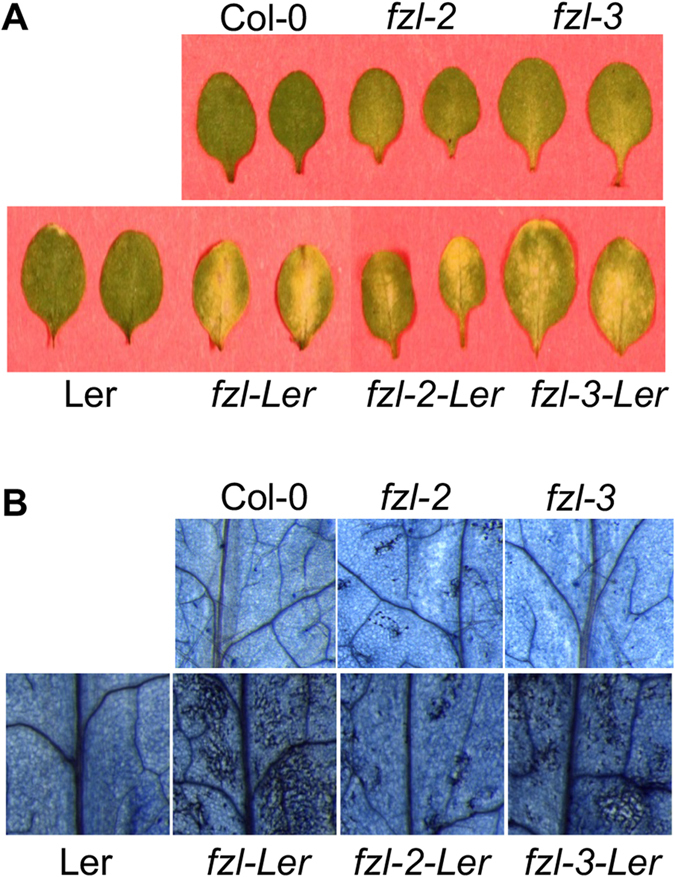
fzl-conferred cell death phenotype can be enhanced in the Ler background. The *fzl* alleles in Col-0 were crossed to Ler. The resulting homozygous plants were isolated for cell death analysis. (**A**) Picture of leaves. (**B**) Cell death staining with trypan blue dye. These experiments were repeated two times with similar results.

We obtained two additional mutant alleles, *fzl-2* (SALK_118335) and *fzl-3* (SALK_152584) in Columbia-0 (Col-0) background (Fig. 1A). Compared with Col-0 leaves, leaves of *fzl-2* and *fzl-3* mutants appeared to be paler but did not show obvious cell death (Fig. 2). The *fzl-2* and *fzl-3* alleles showed either higher SA accumulation nor enhanced resistance to *P. syringae* infection, compared with Col-0 (data not shown). However, when infected with the oomycete pathogen *Hyaloperonospora arabidopsidis (Hpa*) isolate Noco2, these mutants demonstrated higher resistance than Col-0 (Fig. 1E). Ler and *fzl-Ler* plants cannot be tested in this experiment because the Ler background confers hyper-resistance to *Hpa* Noco2. Thus these data suggest that *FZL* is a negative regulator of disease resistance to *P. syringae* and *Hpa* strains and cell death.

**Figure 3:**
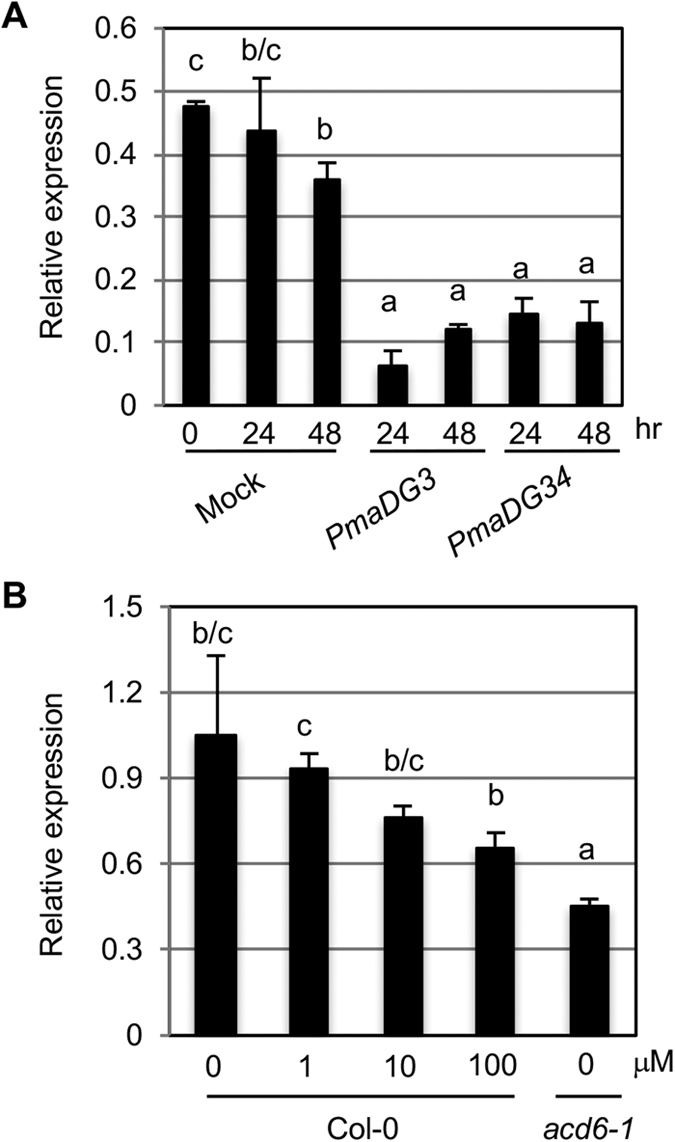
Expression of FZL is suppressed by P. syringae infection and by SA activation. (**A**) *P. syringae* infection suppresses *FZL* expression. Plants were infected by the virulent strain *P. syringae* pv. *maculicola* ES4326 DG3 (PmaDG3) or the avirulent strain PmaDG34 (expressing the avirulence effector *avrRpm1*)^66^. (**B**) Activation of SA signaling suppresses *FZL* expression. Col-0 plants were treated with 100 μM BTH for 24 hrs. qRT-PCR was performed with RNA extracted from the treated and control plants. These experiments were repeated two times with similar results. Different letters indicate significant difference among the samples (P < 0.01).

Consistent with the defense role of *FZL*, we found that expression of *FZL* was suppressed by *P. syringae* infection (Fig. 3A). To further test if *FZL* expression can be affected by SA, we treated plants with an SA analog, benzo (1, 2, 3) thiadiazol-7-carothioic acid (BTH), which was shown to activate similar defense responses in plants as SA but without the toxicity caused by SA^22^,^23^,^24^. Indeed, expression of *FZL* was lower in BTH treated plants in a dosage-dependent manner (Fig. 3B). We also observed a lower expression of *FZL* in the *accelerated cell death 6-1 (acd6-1*) mutant that shows constitutive defense and high SA levels (Fig. 3B and refs 25,26). Thus these results suggest a negative feedback regulation of *FZL* by SA.

### The cell death phenotype of *fzl* mutants can be enhanced by the Ler background

We were intrigued by the effects of different genetic backgrounds on cell death formation in the *fzl* mutants. We confirmed that the two Col-0 *fzl* mutants were allelic to *fzl-Ler* by crossing *fzl-Ler* with *fzl-2* or *fzl-3*. The resulting F_1_ plants demonstrated strong cell death in leaves as *fzl-Ler* (data not shown). Such phenotypic discrepancy in the *fzl* alleles suggests genetic background could affect cell death formation mediated by *FZL* in Arabidopsis. To test this possibility, we crossed *fzl-2* and *fzl-3* with Ler and found that *fzl-2-Ler* and *fzl-3-Ler* homozygous plants showed more severe cell death than the original mutants in Col-0 background (Fig. 2).

### *fzl-*conferred cell death phenotype is regulated by SA

Some lesion mimic mutants showed SA-dependent cell death^9^. To test if *fzl-*conferred cell death phenotype is also SA-dependent, we conducted a mutant analysis. Given the complexity of genetic background on the manifestation of *fzl-*conferred cell death (Fig. 2), it is important to use the same genetic background in the mutant analysis. While most SA mutants are in Col-0 background^20^,^25^,^27^,^28^, the *eds1-2* mutant is a Ler allele and is impaired in major SA accumulation^29^. Thus we crossed *fzl-Ler* with *eds1-2* and found that the double mutant *fzl-Ler eds1-2* had much suppressed but not completely abolished cell death and restored wt-levels of SA, compared with *fzl-Ler* (Fig. S2). Our data are largely consistent with the results from a previous study^19^, supporting a major role of SA in regulating cell death in *fzl-Ler*.

**Figure 4:**
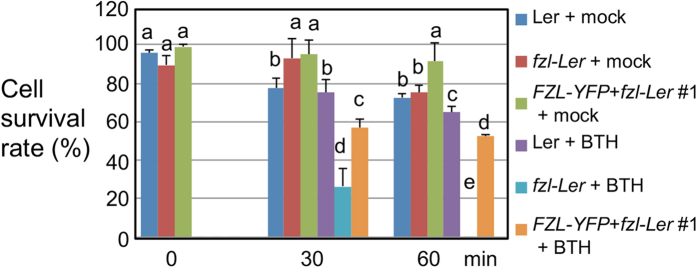
Cell death conferred by *fzl-Ler* can be enhanced by SA treatment. Protoplasts were prepared from 21-day old plants and treated with 100 μM BTH or water. Cell survival at the indicated times was assessed with fluorescein diacetate staining and recorded by fluorescence microscopy. The survival rate was calculated as the follow: No. of living protoplasts/no. of total protoplasts *100. Different letters indicate significant difference among the samples at the same time point (P < 0.01; Mann-Whitney test). This experiment was repeated three times and similar results were obtained.

To further test the role of SA in regulating cell death in *fzl* mutants, we used a single-cell system. We treated protoplasts of Ler, *fzl-Ler* and a complementation line (*FZL-YFP* + *fzl-Ler #1*) with BTH. Protoplast survival rate was recorded by using fluorescein diacetate (FDA) staining. We found that mock treatment did not induce much difference in cell death in the protoplasts. Upon BTH treatment, *fzl-Ler* protoplasts showed much faster and more cell death (Fig. 4). By 1 hr of BTH treatment, all *fzl-Ler* cells were dead while most Ler and *FZL-YFP/fzl-Ler* cells remained alive. Similarly, protoplasts of the two Col-0 *FZL* alleles, *fzl-2* and *fzl-3*, also showed enhanced cell death with BTH treatment, compared with Col-0 control (Fig. S3). Since BTH treatment of wild type (wt) protoplasts did not activate cell death, these results suggest that SA signaling works together with *FZL* to control cell death.

### *fzl-Ler* shows increased autophagosomes and ROS accumulation besides altered chloroplast morphology

**Figure 5:**
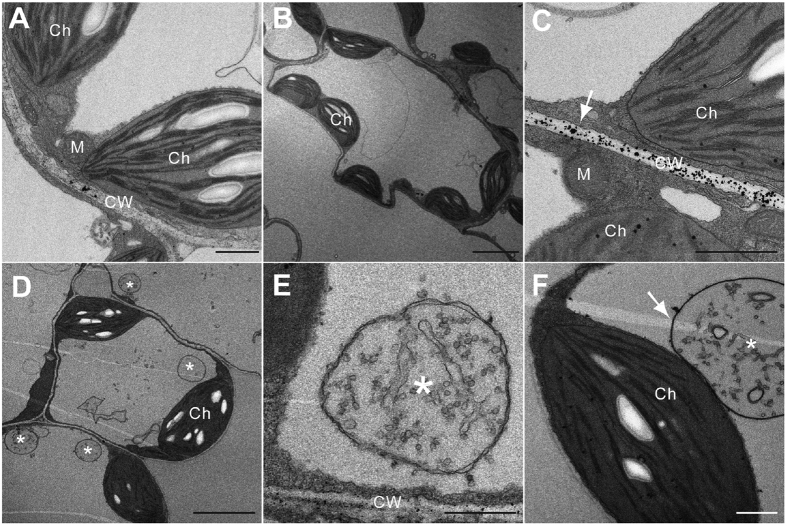
TEM analyses of chloroplast morphology, autophagosome, and ROS deposition. The fourth to sixth leaves of each genotype were collected and cut into 1 × 2 mm sections, which were incubated with freshly prepared 5 mM CeCl_3_ in 50 mM MOPS at pH 7.2 or MOPS only for 1 hr. The samples were then fixed and processed for TEM imaging. At least three different leaf samples of each genotype were used in each experiment. Images represent typical observations in two independent experiments. (**A**,**B**) Images of Ler cells. Note that all organelles are cerium-free. (**C**) Image of *fzl-Ler* cells to show electron-dense cerium deposits mainly on the cell wall. (**D**–**F**) Images of *fzl-Ler* cells to show autophagosome accumulation. Arrows in panels (**C**,**F**) indicate cerium deposits. The size bar in panels (**A**,**E**,**F**) is 1 μm, in panel (**B**,**D**) is 5 μm, and in panel (**C**) is 500 nm. *autophagosome; Ch, chloroplast; CW, cell wall; and M, mitochondrion.

Previous studies showed that *fzl* mutations caused abnormal chloroplast morphology^19^,^21^. We confirmed this phenotype with transmission electron microscopy (TEM), using the fourth to sixth leaves of 21-day old plants. The chloroplast of *fzl-Ler* cells showed reduced thylakoid stacks but more elongated thylakoid grana, compared with that of Ler cells (Fig. 5 and S4). The average number of chloroplasts in *fzl-Ler* cells is 3.35 ± 0.16 (standard error of the mean; n = 78), which is significantly lower than that in Ler cells (4.76 ± 0.40; n = 60). No major difference was observed in the mitochondrial morphology.

**Figure 6:**
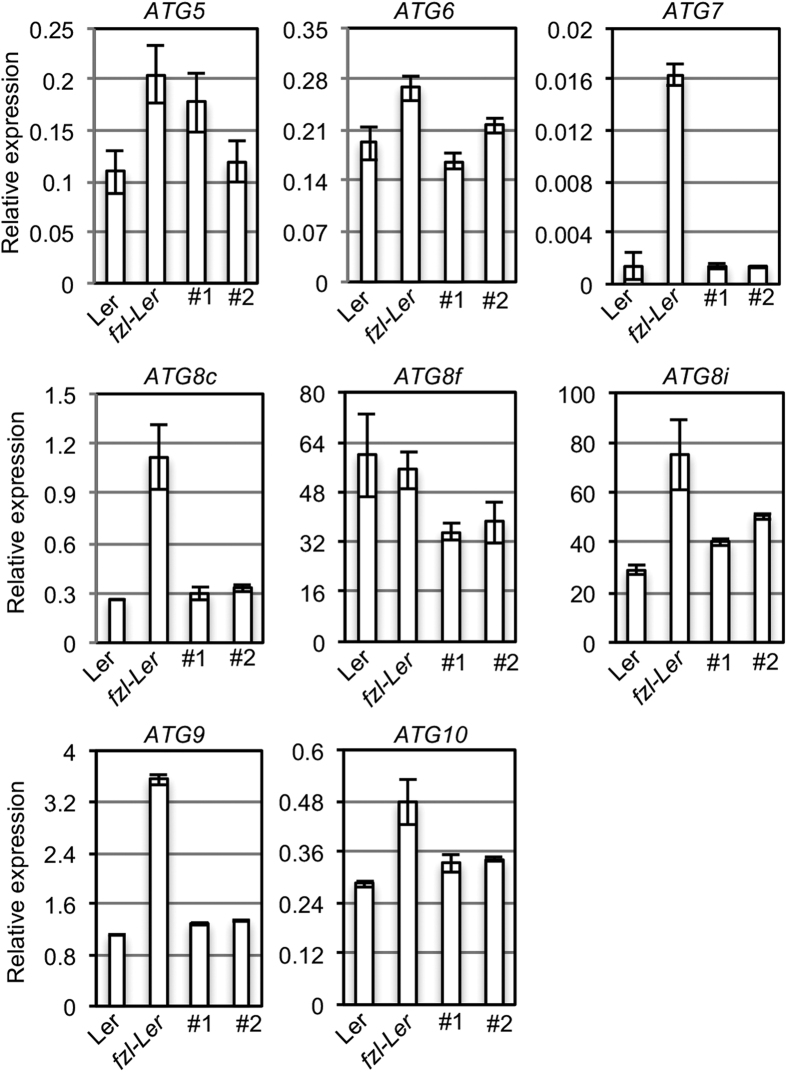
Expression analysis of the *ATG* genes by qRT-PCR. Total RNA was extracted from Ler, *fzl-Ler*, and two complementation lines (*FZL-YFP* + *fzl-Ler* #1 and #2) and used for qRT-PCR analysis of gene expression. These experiments were repeated two times with similar results.

Interestingly, we noticed the accumulation of vesicles in the cytoplasm of *fzl-Ler* cells, some of which were adjacent to the chloroplast in a cell (Fig. 5D–F). These vesicles have a double membrane surrounding the vesicle content, resembling autophagosomes that represent the ancient vesicle mechanism to engulf and deliver cytoplasm content for degradation^3^,^30^. Leaf cells of *Ler* and *fzl-Ler/FZL-YFP* plants did not have obvious autophagosome accumulation (Fig. 5A,B and data not shown). To further confirm the activation of the autophagy pathway in *fzl-Ler,* we measured expression of several *ATG* genes (*ATG5, 6, 7, 8c, 8f, 8i, 9,* and 10) that are important for the formation of autophagosomes. We found that expression of *ATG7, 8C, 8i*, and *9* in *fzl-Ler* was at least two-fold higher than that in *Ler* and *fzl-Ler/FZL-YFP* plants (Fig. 6). Thus cell death formation in *fzl-Ler* likely involves the activation of autophagy.

Oxidative bursts lead to the production of reactive oxygen species (ROS), which are important for activation of defense and cell death^31^,^32^,^33^. Autophagy is tightly connected with ROS production by removing damaged proteins due to oxidation and thus affects cell survival^34^,^35^. To see how *FZL* could affect ROS accumulation and localization, we performed histochemical staining using cerium chloride (CeCl_3_), which reacts with H_2_O_2_ to produce electron-dense precipitates of ceriumperhydroxide that can be visualized using TEM^36^,^37^. The fourth to sixth leaves of *fzl-Ler* and Ler plants were fixed in the presence of cerium chloride followed by embedding and sectioning. Analysis of ultra-thin sections of the embedded samples showed abundant electron-dense cerium deposits, an indicator of H_2_O_2_ accumulation, mainly on the cell wall of the *fzl-Ler* mutant (Fig. 5C and S4). Minor cerium deposits were seen in the chloroplast, cytoplasm, and autophagosome membrane adjacent to the chloroplast (Fig. 5C and F, arrows). However, Ler and *fzl-Ler/FZL-YFP* plants generally lack such dark deposits (Fig. 5A,B and data not shown). These results indicate that *fzl-Ler-*conferred cell death is associated with increased ROS production and autophagosome formation.

### FZL is a chloroplast protein that functions differently from its yeast homolog

FZO1 proteins in yeasts, flies, and mammals are known to localize to the mitochondria and affect mitochondrial fusion^38^. Although being the only close homolog of FZO1 proteins, Arabidopsis FZL was localized to the chloroplast and affected chloroplast morphology. The predicted FZL protein has a potential mitochondrial targeting sequence. Whether FZL could also localize to the mitochondria and regulate mitochondrial function has not been explicitly ruled out. To address this question, we used a single cell system, utilization protoplasts from plants expressing a functional FZL-YFP under the control of the constitutive promoter CAMV 35 S (Fig. 1 and S1B-1D). We first confirmed chloroplast-localization of FZL-YFP, which resided on the outer chloroplast membrane (Fig. S5A and B and^21^). To test if the FZL-YFP protein also resides in the mitochondria in Arabidopsis, we stained protoplasts expressing *FZL-YFP* with the mitochondria-specific dye MitoTracker Red CMXRos. However, we did not observe a co-localization of YFP and MitoTracker red signals (Fig. S5C). Similarly, co-expression of a mitochondrial marker gene tagged with the red fluorescent reporter mCherry^39^ in *FZL-YFP* protoplasts did not reveal a co-localization of mCherry with FZL-YFP (Fig. S6). Thus FZL-YFP is unlikely localized to the mitochondria.

**Figure 7:**
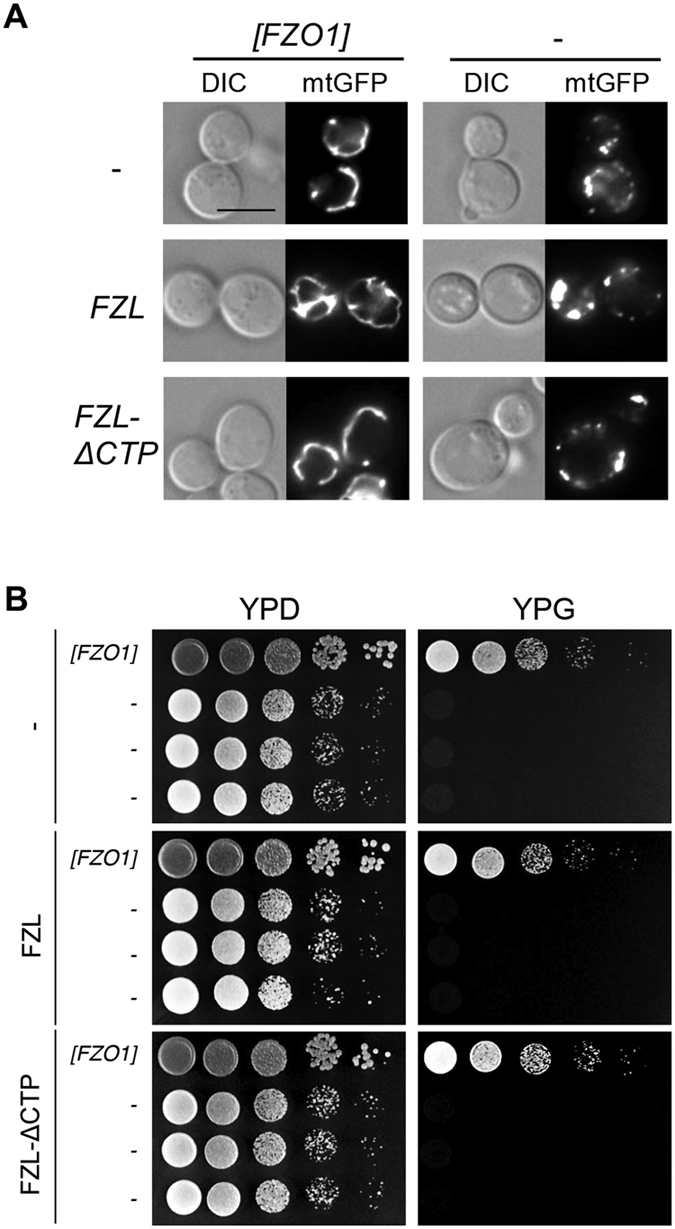
Expression of Arabidopsis *FZL* gene fails to rescue the yeast *FZO1*-lacking mutant. The [*FZO1]* yeast strain has the chromosomal *FZO1* gene deleted and expresses the *FZO1* gene on a plasmid. The minus sign (−) indicates strains not expressing the *FZO1* gene. Both [*FZO1]* and “-” yeast strains expressed mitochondria-targeted GFP (mtGFP). In a plasmid shuffling experiment yeast strains were generated to carry an empty vector, the vector with the full-length *FZL cDNA (FZ*L) or an *FZL* variant with the chloroplast transit peptide-coding sequence being deleted (*FZL-ΔCTP*). (**A**) Mitochondrial morphology. Note there was no rescue of the *FZO1* lacking yeast mutant by Arabidopsis *FZL* or *FZL-ΔCTP.* DIC: Differential interference contrast (DIC). (**B**) Yeast growth assay. Fermentable YPD medium and non-fermentable YPG medium were used to grow yeast strains. These experiments were repeated two times with similar results.

Although not detected in the mitochondria of Arabidopsis cells, it is still possible that the Arabidopsis *FZL* gene shares conserved function with its yeast homolog *FZO1* because the two proteins share significant similarity with 23% identity^16^,^17^,^18^. To test this, we expressed the full-length *FZL* cDNA and a truncated version with a deletion of the DNA fragment encoding the chloroplast transient peptide (*FZL-ΔCTP*) in yeast strains with or without the *FZO1* gene. These yeast strains also expressed mitochondria-targeted GFP, mtGFP^40^. GFP fluorescence images showed typical wt-like tubular mitochondria in the strains expressing *FZO1* (Fig. 7A left). However, the lack of *FZO1* led to fragmented mitochondria (Fig. 7A right). Expression of Arabidopsis *FZL* or *FZL-ΔCTP* failed to rescue the mitochondrial defect in the *FZO1-*deletion yeast strain.

Yeast cells lacking *FZO1* eventually lose mitochondrial DNA and can only grow on the fermentable YPD medium (containing glucose) but not on the non-fermentable YPG medium (containing glycerol) (Fig. 7B and ref. 17). Consistent with results shown in Fig. 7A, only the strains expressing *FZO1* but not the *FZO1*-lacking strains that expressed Arabidopsis *FZL* or *FZL-ΔCTP,* were able to grow on YPG (Fig. 7B). Together these data further support that the Arabidopsis *FZL* gene has evolved new function different from its yeast homolog *FZO1*.

## Discussion

The *FZL* gene was previously reported to affect chloroplast morphology and cell death in Arabidopsis^19^,^21^. We identified an *fzl* mutant based on its lesion mimic phenotype. Further characterization of *fzl* mutants showed that besides altered chloroplast morphology and cell death, disruption in the *FZL* gene led to more resistance to both bacterial and oomycete pathogens, the activation of autophagy and ROS pathways. Cell death conferred by *fzl* is SA-dependent and can be further exacerbated by SA. While the lack of *FZL* led to increased SA accumulation, more SA could in turn suppress expression of *FZL*. Protein co-localization study and yeast complementation test suggest that the Arabidopsis *FZL* gene may have evolved new function different from its homologs in yeast, flies, and mammals. This new function of *FZL* likely involves the autophagy and ROS pathways, leading to the regulation of defense and cell death in Arabidopsis.

The yeast *FZO1* is the founding member *of FZO-*like genes and its protein product was shown to localize to the mitochondria and affect mitochondrial function^16^,^17^,^18^. The mitochondria are dynamic organelles with constant movement inside of the cell along with frequent fusion and fission. Such dynamic behavior of the mitochondria is critical for mitochondrial function in many biochemical reactions, energy production, and cellular respiration^38^. The yeast *fzo1* mutant showed highly fragmented mitochondria and instable mitochondrial DNA. Later studies with disrupted *FZO* homologs in flies, worms and mammals showed similar mitochondrial fragmentation^12^,^13^,^14^,^15^,^18^. These studies indicate that *FZO* genes regulate mitochondrial fusion, disruption of which could lead to disease in some organisms^41^,^42^,^43^,^44^,^45^. *FZL* is the only Arabidopsis gene that shares significant homology to the *FZO* genes in yeast, flies, and mammals. It was originally hypothesized to function similarly to these other *FZO* genes in regulating mitochondrial function. However two previous studies^19^,^21^ and our data presented here support new function of the Arabidopsis *FZL* gene in the chloroplast. First, while mutations in *FZL* led to cell death in Arabidopsis, the yeast *fzo1* mutant did not show a cell death phenotype (this study and refs 19,46). Second, it is the morphology of the chloroplast, not the mitochondria, that is altered in *fzl-Ler* when compared with Ler (Fig. 5, S4, and refs 19,21). Third, the FZL protein was localized to the chloroplast but not in the mitochondria of Arabidopsis cells (Figs S5 and S6, and ref. 21). Fourth, Arabidopsis FZL or an FZL variant did not rescue the *FZO-*lacking yeast strain (Fig. 7). Thus the Arabidopsis *FZL* gene likely functions differently from its homologs in yeast, flies, and mammals and Arabidopsis uses an *FZL*-independent mechanism in regulating mitochondrial fusion.

The function of *FZL* is closely related to the chloroplast. Besides its critical role in photosynthesis, the chloroplast has been shown as the primary source of many important defense molecules, such as SA biosynthesis, production of ROS and some secondary compounds^47^,^48^. Like the mitochondria, the chloroplast is also an important player in PCD^49^. Mutations in several genes affect chloroplast-derived metabolites and confer the lesion mimic phenotype. Examples of such mutants include *acd1* (impaired in pheophorbide an oxygenase)^50^, *acd2* (impaired in red chlorophyll catabolite reductase)^44^,^51^, *flu* (impaired in a protein that is a part of a complex inhibiting tetrapyrrole synthesis)^52^,^53^, and the maize mutant *les22* (impaired in uroporphyrinogen decarboxylase)^54^. FZL is an outer-membrane chloroplast protein (Figs S5 and S6, and ref. 21) and likely functions differently from these chloroplast metabolic proteins in affecting PCD. The lack of FZL could cause the change in chloroplast morphology and subsequently affect the function of the chloroplast. Such changes in the chloroplast can activate stress signals, leading to the production of ROS, which in turn could cause the accumulation of damaged proteins and result in cell death. Consistent with the role of autophagy in removing damaged proteins and regulating PCD, we observed increased autophagosomes using high-resolution TEM images and enhanced expression of autophagic genes in the *fzl* mutant exhibiting cell death (Figs 5 and 6). Thus our data support the regulation of autophay and ROS pathways by FZL. Further detailed morphological analysis of autophagosomes in *fzl-Ler* and genetic analysis of *fzl* mutants and other mutants impaired in autophagy and ROS pathways could contribute to a better understanding of FZL function.

Another factor affecting FZL function is SA as demonstrated by these supporting data: (1) *fzl-Ler* accumulates higher SA levels, associating with more cell death and disease resistance, compared to Ler (Fig. 1 and S1); (2) The high SA level in *fzl-Ler* is suppressed by a normal FZL gene and by the *eds1-2* mutation. Such suppression in SA accumulation is associated with reduced cell death and defense (Fig. 1, S1, and S2); (3) SA treatment activates cell death in *fzl* mutants but not in wt alone (Fig. 4 and S3) and (4) expression of *FZL* is suppressed by high SA levels and defense activation (Fig. 3). Together these observations suggest a negative feedback loop formed between FZL and SA in regulating cell death and defense. The FZL-SA signaling loop likely also involves the ROS and autophagy pathways. To support this notion, ROS bursts are known to trigger SA production and signaling and in turn SA signal activation can induce more ROS bursts^33^. A similar interplay was found with autophagy and SA^3^,^11^,^55^,^56^. Thus FZL may normally function be to inhibit the activation of the signaling cascade involving ROS, SA and autophagy. In the absence of FZL, the activation of this signaling event leads to degradation of the chloroplast (the source of some pro-death signals) and eventually cell death. Indeed, we observed reduced numbers of chloroplasts in addition to cell death in *fzl-Ler*. While we know disrupting SA could suppress *fzl-*conferred phenotypes, it would be interesting to further investigate how disrupting ROS and autophagy pathways could interfere with *fzl-*conferred phenotypes in future studies.

Besides ROS, SA, and autophagy, *fzl*-conferred cell death can be affected by other factors. The *fzl-2* and *fzl-3* alleles in Col-0 background demonstrated minor defense and no cell death while the *fzl-ler* allele in Ler background showed strong cell death and defense phenotypes (Figs S1 and 2). We showed that the Ler background can enhance cell death in *fzl-2* and *fzl-3* mutants. Consistent with the influence of genetic background on *fzl-*conferred cell death, we observed residual cell death when the SA mutant *eds1-2* in Ler background was crossed into *fzl-ler* while another study showed a complete suppression of cell death in *fzl-Ler* by a different *eds1* mutant (Fig. S2 and ref. 19). Such discrepancy could be due to genetic background and/or the growth environment in different laboratories. Developmental stages could also affect *fzl*-conferred cell death (Fig. S1A). Together these observations suggest that additional molecules existing under certain conditions could affect FZL function.

The roles of ROS and SA in plant defense against pathogens have been relatively well understood. Pathogen infection and activation of defense signaling are known to induce autophagy^5^,^6^,^7^. However how autophagy regulates plant innate immunity remains unclear. While they all had much reduced autophagosomes and increased cell death, *atg* mutants showed opposing effects on disease resistance. One *atg* mutant, *atg6,* was shown to be more susceptible to biotrophic pathogens^57^,^58^. Consistent with *atg6*-conferred disease susceptibility, studies on *fzl* and the mutant defective in glyceraldehyde-3-phosphate dehydrogenase (GAPDH) showed an association of constitutive autophagy with enhanced disease resistance against biotrophic pathogens. On the other hand a group of *atg* mutants (*i.e. atg*2, 5, 10, and 18a) was shown to be more resistant to biotrophic pathogens^56^,^59^,^60^,^61^. Plants usually respond to biotrophic and necrotrophic pathogens with opposing defense phenotypes^62^. Consistent with this idea, these *atg* mutants were more susceptible to necrotrophic pathogens. Interestingly another group of *atg* mutants (i.e. *atg7* and 9) was shown to be more susceptible to both biotrophic and necrotrophic pathogens. It appears that there is a lack of consistent association between the number of autophagosome formation and plant defense. These seemly controversial data could suggest that different *ATG* genes are differentially required by pathogens of different lifestyles and the severity of cell death is not always coupled with the level of disease resistance in plants. Thus the mechanism of autophagy signaling in defense, in particular its connection with FZL function, still remains to be elucidated.

Taken together the *fzl-ler* mutant exhibits hallmarks of lesion mimic mutant phenotypes, including cell death, increased accumulation of defense-related molecules (i.e. ROS, SA and defense gene transcripts). We report that the activation of the autophagy pathway in this mutant is a possible mechanism leading to cell death in the plant. Data from this and other studies clearly support that the *FZL* gene of Arabidopsis has evolved new function different from its homologs in yeast, flies, and mammals. This new function lies in the regulation of chloroplast morphology and function, activation the signaling cascade involving ROS, SA and autophagy. The FZL-regulated processes ultimately affect plant growth, development, and response to pathogen attacks. Although we are still far from a complete understanding of the molecular mechanism by which FZL regulates innate immunity, *FZL* and its related genes can be used as excellent resources to uncover mechanisms of PCD and disease resistance. These genes can also be used as powerful tools to manipulate plant defense response in order to achieve a broad spectrum of disease resistance in plants.

## Methods

### Plant Materials

Most Arabidopsis plants used in this research were grown in growth chambers with a 12 hr light/12 h dark cycle, light intensity at 200 μmol m^−2^ s^−1^, 60% humidity, and 22 °C. For protoplast isolation, plants were grown under lower light intensity (100 μmol m^−2^ s^−1^) with other conditions the same. The *fzl-Ler* and *eds1-2* mutants were previously described^19^,^29^. The *fzl-2* (SALK_118335) and *fzl-3* (SALK_152584) mutants were obtained from the Arabidopsis Biological Resource Center.

### Protoplast Analyses

Arabidopsis protoplasts were isolated from leaves of 21-day old plants following the tape sandwich technique described previously^63^ with few modifications. Briefly, the lower epidermal surface cell layer was peeled away from leaves using plastic tape. Fifteen peeled leaves were transferred to a Petri dish containing 10 ml of enzyme solution (20 mM MES (pH 5.7), 1% (w/v) cellulase R10, 0.25% (w/v) macerozyme R10, 0.4 M mannitol, 20 mM KCl, 10 mM CaCl_2_, 0.1% (w/v) BSA). Leaves were gently agitated in the dark for 60 to 120 min till the protoplasts were completely released into the solution. Protoplasts were centrifuged three minutes at 100 × g, washed twice with 10 ml of pre-chilled modified W5 solution (2 mM MES (pH 5.7)), 154 mM NaCl, 125 mM CaCl_2_, 5 mM KCl, 5 mM glucose, and incubated on ice for 30 min. Protoplasts were then centrifuged and resuspended to a final concentration of 5 × 10^5^ cells/ml in modified MMG solution (4 mM MES (pH 5.7), 0.4 M mannitol, 15 mM MgCl_2_) for further experiments.

For co-localization studies, protoplasts expressing *FZL-YFP* were stained with the mitochondria-specific dye MitoTracker Red CMXRos (Molecular Probes) or transfected with a mitochondrial marker gene tagged with the red fluorescent reporter gene mCherry^39^. Images were captured using a confocal laser scanning microscope (Leica TCS SP2 AOBS) and analyzed using the Imaris software (version 7.0.0).

### Complementation Tests

The complementation construct for *fzl-Ler* was made by fusing *FZL* cDNA translationally at the 3′ end with the *YPF* reporter and the chimeric gene was expressed under the control of the *CAMV 35 S* promoter. This construct, named *FZL-YFP*, was used to transform *fzl-Ler*. At least 15 independently transformed lines were obtained and they all showed abolished cell death phenotype. Primers used for making the construct were listed in Table S1.

In order to complement the yeast *FZO1*-lacking mutant, we cloned the full-length *FZL* cDNA and a truncated version with a deletion of the sequence encoding the chloroplast transient peptide (*FZL-ΔCTP*) in the pYX122 vector under the control of the constitutive TPI promoter. These constructs and the empty vector were introduced into a yeast strain that has the chromosomal copy of the *FZO1* gene deleted and carries a copy of the *FZO1* gene on a plasmid with a *URA3* marker. Upon growth on the 5-FOA medium, the *URA3* plasmid was counter-selected against and thereby the *FZO1* gene was eliminated in yeast cells, thus creating *FZO1*-lacking yeast mutant strains. Primers used for making yeast-complementation constructs were listed in Table S1.

### Pathogen Infection

Bacterial culture and preparation of *P. syringae* strains were conducted as described^64^. The fourth to sixth leaves of 21-day old plants were infiltrated with *P. syringae*-containing solution, using a 1 ml needleless syringe. Infected leaf discs were collected three days later for bacterial growth assay. The infection with *Hyaloperonospora arabidopsidis* isolate Noco 2 was conducted with 7-d old seedlings as previously described^64^.

### Cell Death Analyses

The fourth to sixth leaves from each genotype were stained with trypan blue for cell death visualization, according to a published protocol^27^. Photographs of the stained leaves were taken with a CCD camera (cool Snap HQ^2^, Photometrics, USA) connected to a dissecting microscope (Leica M205 FA, Leica Microsystems, Germany). At least three leaves from different plants of each genotype were stained and examined for cell death.

To assess cell death in protoplasts, 80 μl of protoplasts at 5 × 10^5^/ml for each genotype were treated with 100 μM of benzo (1,2,3)-thiadiazole-7-carbothioic acid (BTH, a kind gift from Robert Dietrich, Syngenta) or water (mock). Three replicates for each genotype were used in each experiment. Protoplasts were collected at the indicated times and were stained with fluorescein diacetate (FDA) (Sigma-Aldrich Co. LLC, St. Louis MO) for cell viability test, using 2 μl of 5 mg/ml FDA per sample for 2 min in darkness. Living cells gave out green fluorescence that was detected with fluorescence microscopy. The cell survival rate was calculated based on the ratio between living cells and the total number of protoplasts in a sample.

### RNA Analysis

The fourth to sixth leaves of 21-day old plants were harvested for RNA extraction followed by qRT-PCR analysis as previously described^65^. Primers used for qRT-PCR were listed in Table S1.

### SA Measurement

Free and total SA (glucosylated SA) were extracted from 21-day old plants and quantified with a high-performance liquid chromatography (HPLC) instrument as previously described^25^,^28^.

### H_2_O_2_ Localization by Cerium Chloride Staining

The localization of H_2_O_2_ by cerium chloride staining was described previously^66^. The fourth to sixth leaves of 21-day-old plants were cut into 1 × 2 mm pieces, which were first incubated with freshly prepared 5 mM CeCl_3_ in 50 mM 3-(N-morpholino) propanesulfonic acid (MOPS) at pH 7.2 for 1 h. Control samples were incubated in MOPS buffer only. The treated leaf sections were further fixed in 2.5% (v/v) glutaraldehyde and 2% (v/v) paraformaldehyde in 0.1 M cacodylate buffer (pH 7.2–7.4) and embedded in Epon 812 resin (Electron Microscopy Sciences). Ultra-thin sections (90 nm) were used for observation with a transmission electron microscope (JEM-1400, JEOL, Tokyo, Japan) at an accelerating voltage of 120 kV.

## Additional Information

**How to cite this article**: Tremblay, A. *et al.* A Role of the *FUZZY ONIONS LIKE* Gene in Regulating Cell Death and Defense in Arabidopsis. *Sci. Rep.* **6**, 37797; doi: 10.1038/srep37797 (2016).

**Publisher's note:** Springer Nature remains neutral with regard to jurisdictional claims in published maps and institutional affiliations.

## Supplementary Material

Supplementary Information
