# Supplementary material for: A Role of the FUZZY ONIONS LIKE Gene in Regulating Cell Death and Defense in Arabidopsis
Source: Sci Rep. 2016 Nov 29;6:37797. doi: 10.1038/srep37797 (PMC5127180; doi:10.1038/srep37797)
Supplement: Supplementary Information [file srep37797-s1.pdf]

SUPPLEMENTAL FIGURES AND TABLE

A Role of the *FUZZY ONIONS LIKE* Gene in Regulating Cell Death and Defense in Arabidopsis

Arianne Tremblay[1*+], Savanna Seabolt[1*], Hongyun Zeng[2], Chong Zhang[1], Stefan Böckler[3], Dominique N. Tate[1&], Vy Thuy Duong[1], Nan Yao[2], and Hua Lu[1#]

[1] Department of Biological Sciences, University of Maryland Baltimore County, 1000 Hilltop Circle, Baltimore, MD 21250, USA.

[2] State Key Laboratory of Biocontrol, Guangdong Provincial Key Laboratory of Plant Resources, School of Life Sciences, Sun Yat-sen University, Guangzhou 510275, P.R. China

[3] Institut für Zellbiologie, Universität Bayreuth, Bayreuth 95440, Germany

[*] These authors contributed equally to this work.

[+] Current address: AgBio Division, Intrexon Corporation, 1920 5th street, Davis, CA 95616

[&] Current address: Department of Plant Pathology, 201 Kottman Hall, 2021 Coffey Road, Columbus, OH 43210

[#] **Correspondence author:** Hua Lu, Department of Biological Sciences, University of Maryland Baltimore County, 1000 Hilltop Circle, Baltimore, MD 21250. Tel: 410-455-5972 (O), 410-455-2263 (L), Fax: 410-455-3875. Email: hualu@umbc.edu.

Running title: FZL regulates cell death and defense

Key words: programmed cell death, disease resistance, reactive oxygen species, autophagy, mitochondrial fusion, salicylic acid

SUPPLEMENTAL FIGURE LEGENDS

Figure S1. Complementation of *fzl-Ler*-conferred cell death by *FZL-YFP*. (A) Pictures of Ler and *fzl-Ler* leaves at different developmental stages. (B) Pictures of 21-day old plants. (C) Pictures of leaves from 21-day old plants. (D) Pictures of cell death in the leaves. Leaves were stained with trypan blue to visualize cell death and photographed.

Figure S2. *fzl-Ler*-conferred cell death phenotype is largely *EDS1*-dependent. The *fzl-Ler* mutant was crossed to *eds1-2* (a Ler mutant) to obtain the double mutant. (A) Pictures of 21-day old plants. (B) Cell death stained with the trypan blue dye. Arrows indicate dead cells in *fzl-Ler*. (C) SA quantification by HPLC analysis. These experiments were repeated two times with similar results.

Figure S3. SA treatment enhanced cell death in *fzl* mutants in Col-0 background. Protoplasts were prepared from 21-day old plants and treated with or without 100 μM BTH. The number of living cells was determined by fluorescein diacetate staining and cell survival rate was calculated as the follow: number of living cells/total number of protoplast cells *100. Different letters indicate significant difference among the samples at the same time point ($P<0.01$; Mann-Whitney test).

Figure S4. Pictures of the chloroplast ultrastructure from Ler (A) and *fzl-Ler* cells (B). Ch: chloroplast; CW: cell wall. Bar=1μm.

Figure S5. FZL-YFP localizes to the outer membrane of the chloroplast. Protoplasts were isolated from plants expressing *FZL-YFP* and stained with MitoTracker Red CMXRos for a co-localization study. The cells were imaged by confocal microscopy. Bars = 5 μm. (A) YFP fluorescence. (B) YFP 3D reconstruction. The Imaris software (version 7.0.0) was used for image analysis. (C) YFP and MitoTracker Red fluorescence.

Figure S6. FZL-YFP does not co-localize with the mitochondrial marker mCherry. Protoplasts were isolated from plants expressing FZL-YFP and were further transfected with the mitochondrial marker gene tagged with the mCherry reporter for a co-localization study. (A) YFP fluorescence. (B) mCherry fluorescence. (C) Chloroplast

autofluorescence. (D) Cell morphology viewed with transmitted light. (E) Overlay of YFP and mCherry fluorescence.

Table S1. Primers used in this paper

**Purpose**	**Primer Name**	**Primer Sequence (5' > 3')**
**Complementation**		
Arabidopsis *fzl-Ler*	FZL1forpGR117_F	ATGAGAACTCTAATCTCTCACCG
	FZL1forpGR117_R	GAAGTCTCATCTCGTCTCGTG
Yeast *fzo1*	FZL1forpYX_F	ATGAGAACTCTAATCTCTCACCG
	FZL1forpYX_R	CTAAAGTCTCATCTCGTCTCGT
	FZL1CDS-CTP_F	ATGCAGACTTCTTCTTCTAGGCCG
**Gene Expression**		
ATG5	ATG5_qPCR_for	GTGGCTGGACAAGTTAAGACAGC
	ATG5_qPCR_rev	ACTCAACAGGGCGATTAAGGTACG
ATG6	ATG6_qPCR_for	TTGCTAGAACTCAGACACAGGTTG
	ATG6_qPCR_rev	CGCATGCTTCGTATGCTTCCAC
ATG7	ATG7_qPCR_for	ACGTGGTTGCACCTCAGGATTC
	ATG7_qPCR_rev	ACTAAGAGTTCAACGGCGAGAGC
ATG8c	ATG8C_qPCR_for	TCGCCATGGCTAATAGCTCTTTC
	ATG8C_qPCR_rev	TCAGCTCTCTCTACGATCACTGG
ATG8i	ATG8I_qPCR_for	GGGCGGTCGAAGAAGATGAAATCG
	ATG8I_qPCR_rev	TTTCTCGATGGCAGGCAGATCC
ATG8f	ATG8F_qPCR_for	AACGATAGCGTTGTGCTCTGG
	ATG8F_qPCR_rev	GCCTCTGCCCTTCTCTTCTCTAAG
ATG9	ATG9_qPCR_for	TGATGTTTGTCGTGCCAAAGCG
	ATG9_qPCR_rev	TGCAAACATGGCCTACACCTTC
ATG10	ATG10_qPCR_for	ACCAATCGTTCCCTCCATGGTC
	ATG10_qPCR_rev	AGCGACAAGTAACCTTCCTCCTTC
FZL	FZL-qPCR-forward	TGTTGACACACCTGGGACCAATG
	FZL-qPCR-reverse	TGTACCGGAGAAACGCAACCTC
PR1	PR1_qPCR_for	ACACGTGCAATGGAGTTTGTGG
	PR1_qPCR_rev	TTGGCACATCCGAGTCTCACTG

Figure S1
Tremblay et al

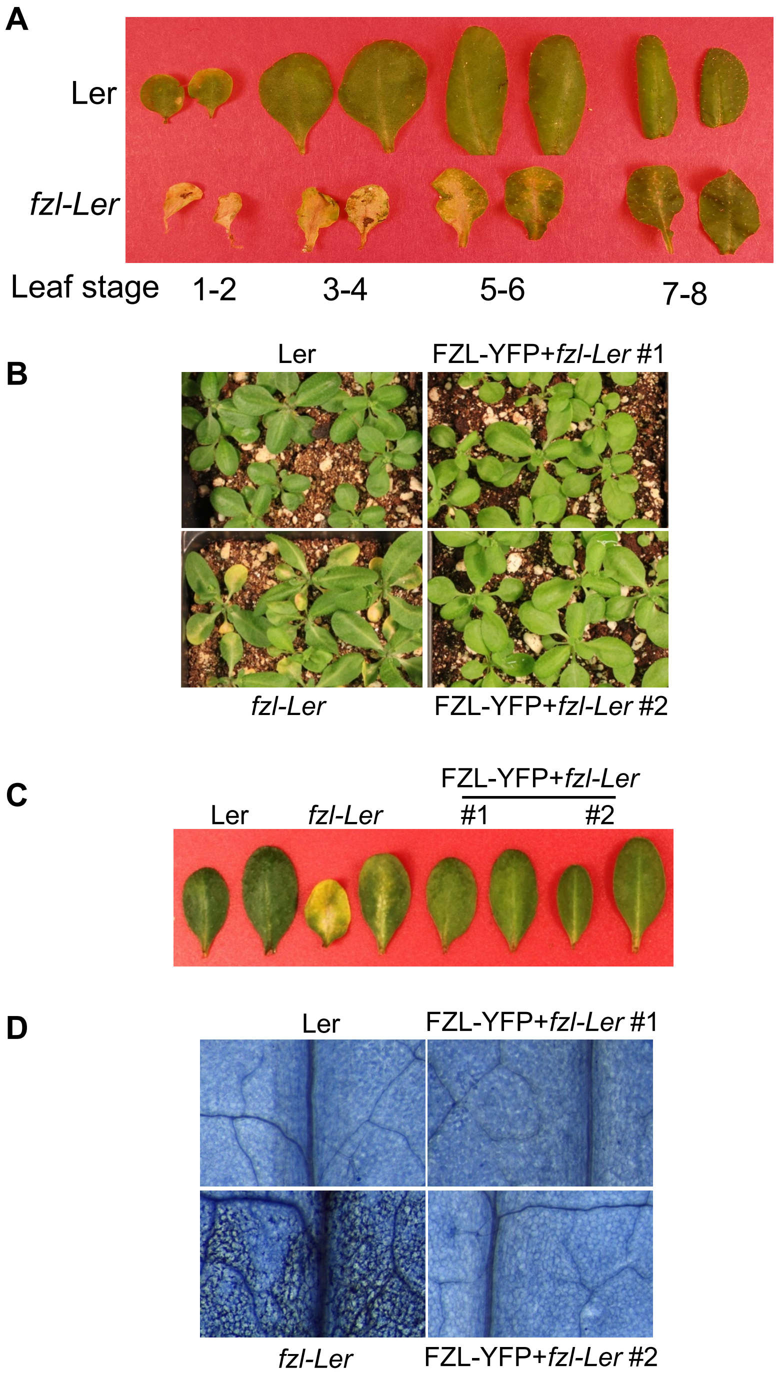

Figure S2
Tremblay et al

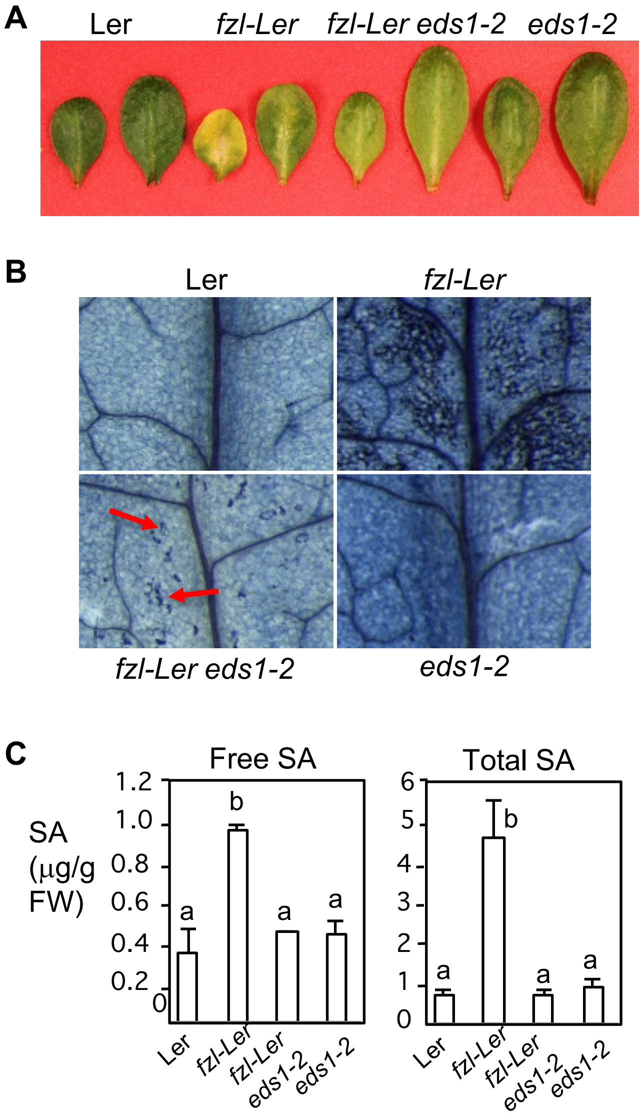

Figure S3
Tremblay et al

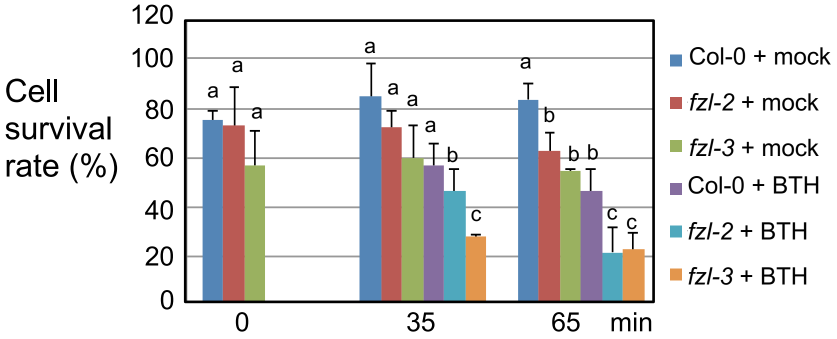

Figure S4
Tremblay et al

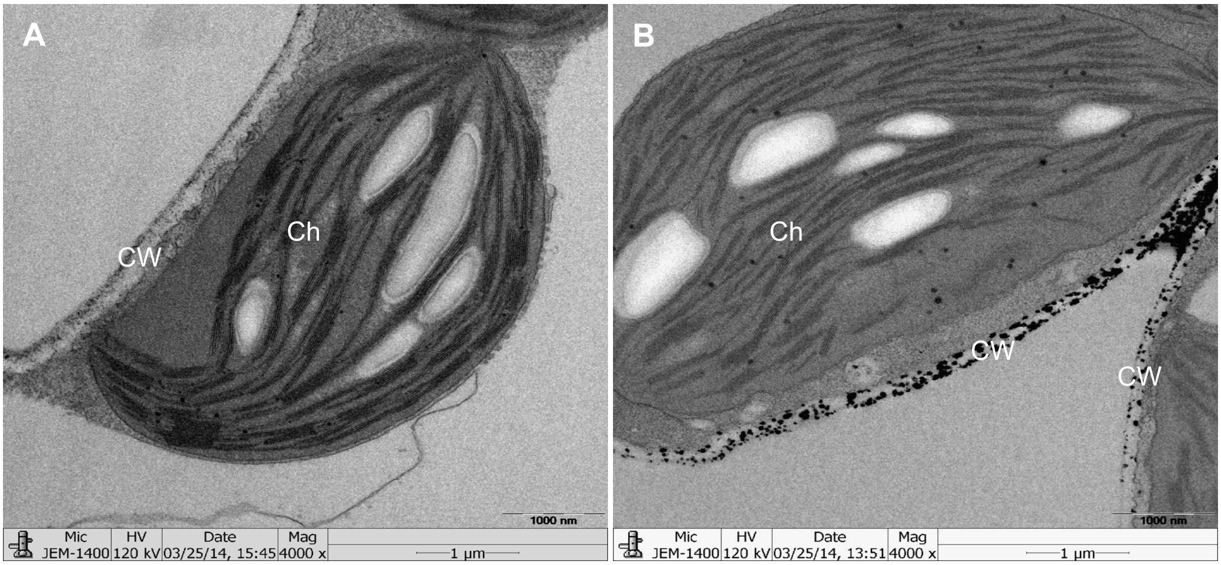

Figure S5
Tremblay et al

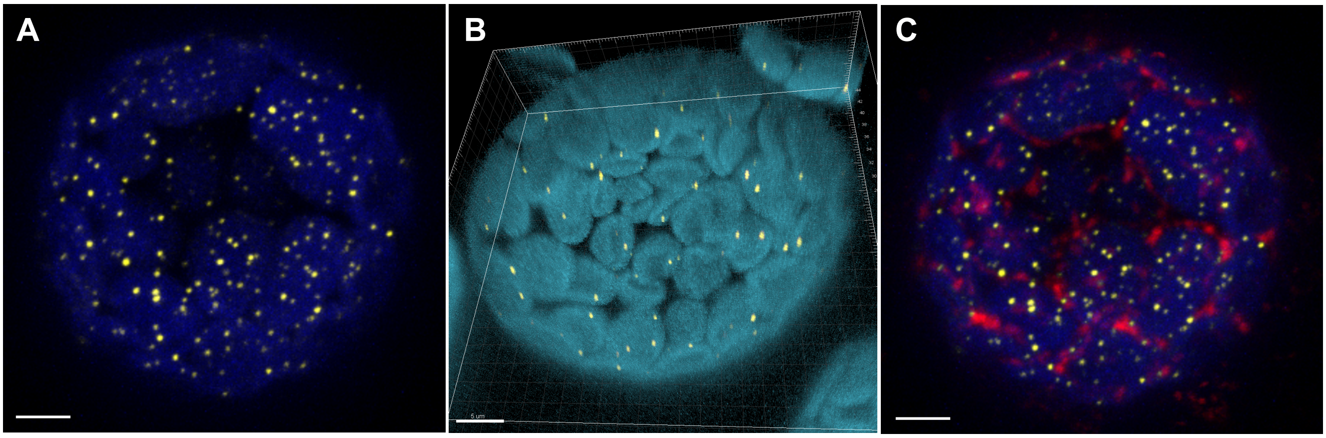

YFP

YFP

YFP and MitoTracker Red

Figure S6
Tremblay et al

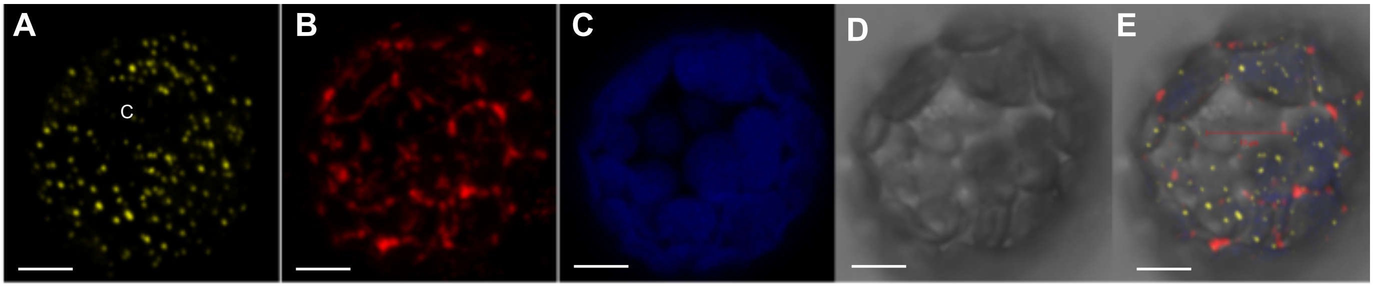

YFP | mCherry | Chloroplast autofluorescence | Transmit light | YFP + mCherry
